# Genetic Diversity among Tropical Provitamin A Maize Inbred Lines and Implications for a Biofortification Program

**DOI:** 10.1556/0806.46.2018.066

**Published:** 2019-03-01

**Authors:** J.P. Sserumaga, D. Makumbi, M.L. Warburton, S.O. Opiyo, G. Asea, A. Muwonge, C.L. Kasozi

**Affiliations:** 1 Cereals Program, National Agricultural Research Organization; National Crops Resources Research Institute, Namulonge, P. O. Box 7084 Kampala, Uganda; 2 International Maize and Wheat Improvement Center (CIMMYT), P.O. Box 1041-00621, Nairobi, Kenya; 3 USDA ARS Corn Host Plant Resistance Research Unit, Mississippi State, MS 39762; 4 Molecular and Cellular Imaging Center, Ohio State University, Columbus, OH, 43210

**Keywords:** maize, inbred, SNP, provitamin

## Abstract

Insights into the diversity and relationships among elite breeding materials are an important component in maize improvement programs. We genotyped 63 inbred lines bred for high levels of provitamin A using 137 single nucleotide polymorphism markers. A total of 272 alleles were detected with gene diversity of 0.36. Average genetic distance was 0.36 with 56% of the pairs of lines having between 0.30 and 0.40. Eighty-six percent of the pairs of lines showed relative kinship values <0.50, which indicated that the majority of these provitamin A inbred lines were unique. Relationship pattern and population structure analysis revealed presence of seven major groups with good agreement with Neighbour Joining clustering and somewhat correlated with pedigree and breeding origin. Utilization of this set of provitamin A lines in a new biofortification program will be aided by information from both molecular-based grouping and pedigree analysis. The results should guide breeders in selecting parents for hybrid formation and testing as a short-term objective, and parents with diverse alleles for new breeding starts as a long-term objective in a provitamin A breeding program.

## Introduction

In sub-Saharan Africa (SSA), maize is the principal staple crop, accounting for an average of 32% of consumed calories in Eastern and Southern Africa, rising to 51% in some countries (Cairns et al. 2012), and it is also a source of income. In the majority of countries in SSA the maize varieties grown are predominantly white grained with low levels of protein, fat, minerals, and micronutrients including provitamin A carotenoids. Although yellow maize is grown and consumed throughout the world including in some countries in SSA, most of these varieties have less than 2 μg g^–1^ of provitamin A carotenoids (Pixley et al. 2013). In developing countries, vitamin A deficiency (VAD) affects up to 140 mil- lion children and pregnant women, and can cause blindness, immune system deficiency, and stunting of growth (West 2002). Developing maize varieties with improved grain quality traits can be achieved through biofortification, which may either involve the use of natural genetic variation existing in the local breeding pool, or by introduction of exotic germplasm. Biofortification of staple crops such as maize with high provitamin A carotenoids through conventional breeding leads to provitamin A enriched maize varieties that could be used to reduce VAD (Adeyemo et al. 2011). Provitamin A maize endosperm contains varying amounts of the provitamin A carotenoids, which include α-carotene, β-carotene, and β-cryptoxanthin, but the concentrations are very low (Adeyemo et al. 2011; Menkir et al. 2017). Some variation for specific carotenoid content has been reported in tropical adapted maize inbred lines (Adeyemo et al. 2011; Menkir et al. 2017). Since the tropical adapted yellow maize varieties grown in SSA contain small amounts of provitamin A carotenoids, there is a need to breed maize for high provitamin A to meet human nutritional requirements (Adeyemo et al. 2011; Brunson and Quackenbush 1962). To achieve this goal, introduction of maize rich in provitamin A carotenoids into tropical maize breeding programs is necessary.

Effective utilization of completely new and uncharacterized maize inbred lines in an existing breeding program requires an understanding of their genetic relationships to identify possible heterotic group placement within the available germplasm. Use of pedigree information is beneficial for grouping of inbred lines when available and when lines are closely related (Liu et al. 2003; Olmos et al. 2014; Sserumaga et al. 2014). Genetic relationships among a group of unrelated lines can also be investigated using molecular genetic markers. Because of low cost per data point, high genomic abundance, locus-specificity, co-dominance, potential for high throughput analysis, and lower genotyping error rates (Rafalski 2002), single nucleotide polymorphism (SNP) markers are a powerful tool for many genetic applications, including genetic diversity studies, linkage and quantitative trait loci (QTL) mapping, and genome-wide association studies (Azmach et al. 2013; Farfan et al. 2015; Suwarno et al. 2015). There is a strong correlation between the molecular marker and pedigree-based distance measures in many plant species, including examples in maize (Liu et al. 2003; Olmos et al. 2014).

In Uganda, like many other countries in sub-Saharan Africa, biofortification has been viewed as a strategy for reducing micronutrient deficiencies. Although there has been great deal of discussion about biofortification as a tool for combating VAD, progress in establishing fortification programs has been slow (Fiedler and Afidra 2010). In response to the emerging demand for biofortified maize, an array of provitamin A maize inbred lines from the International Maize and Wheat Improvement Center (CIMMYT) in Mexico were introduced into Uganda to start a provitamin A carotenoid maize breeding program. There is a need for molecular characterization of these inbred lines to assess their utility in hybrid breeding. The objectives of this study were to assess (i) the level of molecular diversity and population structure among 63 provitamin A maize inbred lines using 137 SNP markers, and (ii) the relationships among the set of 63 provitamin A maize lines for better exploitation in a breeding program.

## Materials and Methods

### Genetic materials and SNP genotyping

Sixty-three maize inbred lines obtained from CIMMYT’s provitamin A carotenoid maize breeding program in Mexico were used in this study (Table S1*). These lines were selected based on high provitamin A content and combining ability. The procedure for developing these inbred lines was described in detail by Pixley et al. (2013). A single seed per inbred line was grown in the greenhouse at the National Crops Resources Research Institute (NaCRRI), Namulonge, Uganda, up to the 3–4 leaf stage. Leaf samples were harvested following the leaf sampling protocol from LGC Genomics (http://www.lgcgroup.com/our-science/genomics-solutions/#.WXpE7ITyu70) using the plant sample collection kit from LGC Genomics (Middlesex, UK). The leaf samples were sent to LGC Genomics in the UK for DNA extraction using LGC’s beadex™ extraction chemistry and genotyping using KASP platform. The 63 samples were genotyped with 142 SNPs and the results were visualized through the SNPviewer software (https://www.lgcgroup.com/products/genotyping-software/snpviewer). The SNPs selected for this study were recommended for routine low-cost genotyping (Semagn et al. 2012) and were well distributed across all chromosomes.

### Data analysis

Gene diversity, polymorphic information content (PIC), allele frequency, and relative kinship were computed using the TASSEL software package (Bradbury et al. 2007). Genetic distance (GD) between lines was calculated based on Rogers distance (Rogers 1972) using PowerMarker version 3.25 (Liu and Muse, 2005). A dendrogram was constructed from the genetic distance matrix using the neighbor-joining technique in PowerMarker, and the resulting trees were visualised using MEGA version 5.0 (Tamura et al. 2011). The model-based clustering approach to analyze population structure was implemented in STRUCTURE software package (Pritchard et al. 2000). To estimate the correct number of clusters in the population of 63 lines using posterior probabilities (qK), a 100,000 burn-in period was used, followed by 100,000 iterations and a model allowing for admixture and correlated allele frequencies with no prior location or population information. At least 10 independent runs of STRUCTURE were performed by setting the number of clusters (K) from 1 to 10, with 10 replicates for each K. The delta K was calculated for each value of K using the Structure Harvester software (Evanno et al. 2005). Structure analysis was run for different values of K. Each inbred line was assigned to a given cluster when the proportion of its genome in the cluster (qK) was higher than a threshold value of 50%. Finally, principal component analysis (PCA) was carried out to illustrate the relationships among the populations based on STRUCTURE results using a three-dimensional plot generated by R package Scatterplot3d (Ligges and Mächler 2002).

## Results

### SNP characterization, genetic distance and relationships

Of the 142 SNPs used for genotyping, 137 showed good amplification in allele calls and were used in further analyses. The 137 SNPs detected a total of 272 alleles, with an average of two alleles detected as expected. Minor allele frequency ranged from 0 to 0.49 (Table S2). Gene diversity in this set of lines ranged from 0 to 0.50 with an average of 0.36 while heterogeneity ranged from 0 to 0.18 with an average of 0.05. The PIC value ranged from 0 to 0.37, with an average of 0.29. Genetic distance between pairs of inbred lines ranged from 0 to 0.54, with an average of 0.36. Most (34.2%) pairs of lines in the present study had genetic distances between 0.35 and 0.40 (Fig. S1). Relative kinship coefficients between pairs of lines ranged from 0 to 0.66, with an average of 0.42 (Figure S2), but most values (86%) fell between 0.35 and 0.50.

### Population structure and principle component analysis

The neighbor-joining (NJ) tree generated from Rogers GD matrix was constructed to gain more insight into the genetic diversity among the set of maize lines. The dendrogram grouped the 63 lines into three major groups with seven subgroups (Fig. 4). The first major group was comprised of four lines. The second major group, which consisted of 35 lines, was split into four subgroups. The third major group was comprised of 24 lines and three subgroups. The model-based approach of STRUCTURE was also implemented to infer population structure for the 63 lines. The output from STRUCTURE based on (LnP(D)) and ΔK suggested the presence of either 2, 5, or 7 subgroups (clusters) among the provitamin A lines (Fig. 2). When K = 7, there was excellent agreement with the seven subgroups of the NJ tree constructed with the genetic distance matrix (Fig. 2c). The membership of the lines in the 7 subgroups were 6% (group 1), 25% (group 2), 16% (group 3), 19% (group 4), 22% (group 5), 3% (group 6) and 6% (group 7) (Fig. 3c).

**Figure 1:**
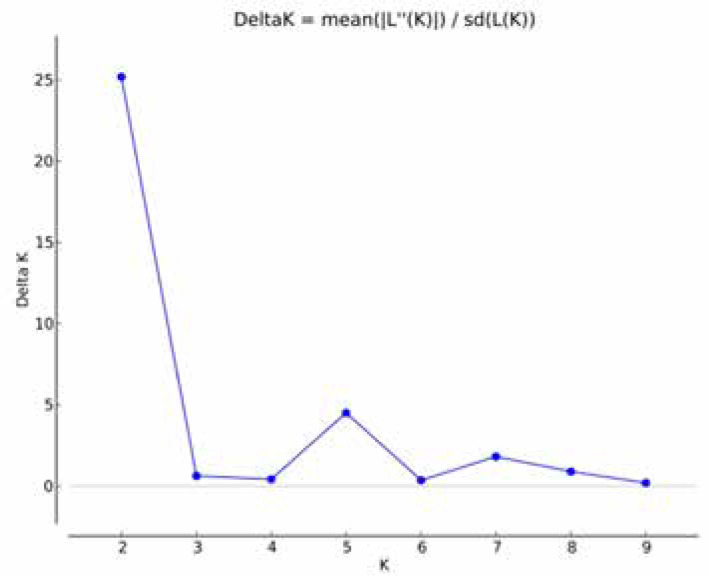
Plot of changes in ΔK value with the number of subpopulations

**Figure 2:**
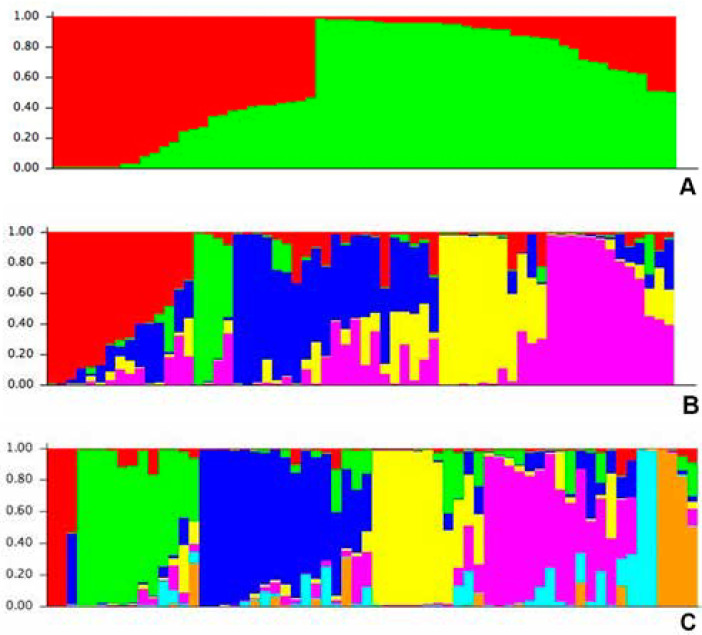
A: Population structure among individuals with ΔK = 2. B: Population structure among individuals with ΔK = 5. C: Population structure among individuals with ΔK = 7

**Figure 3:**
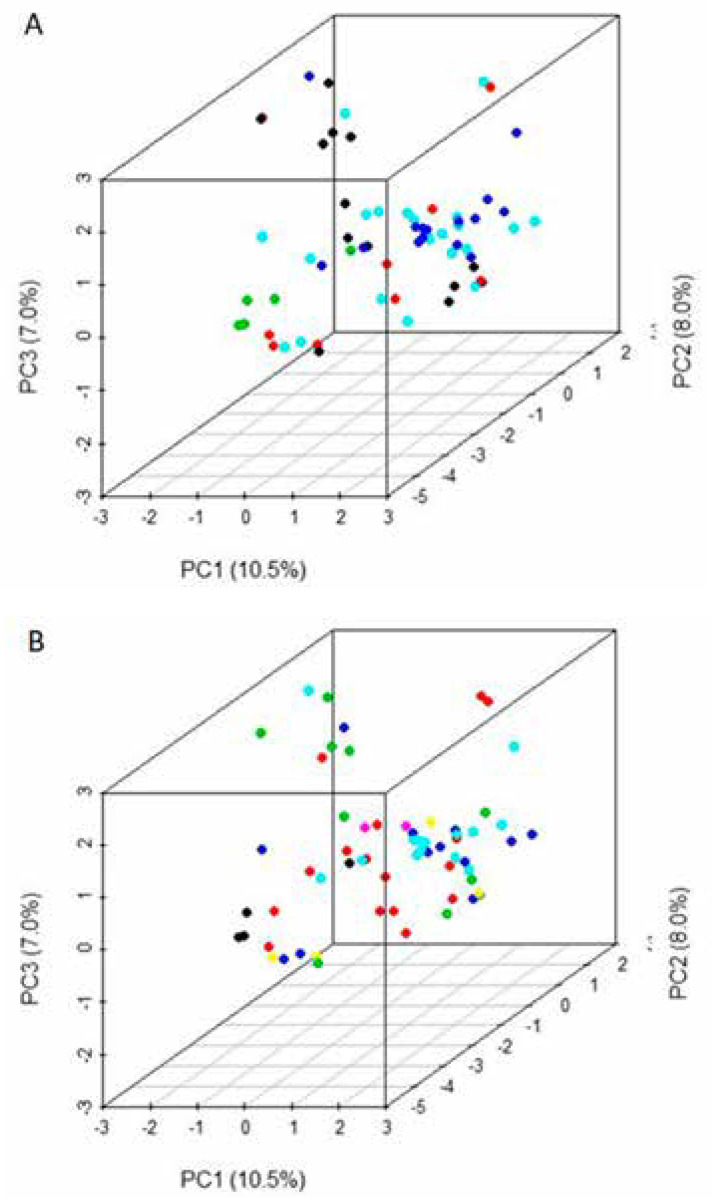
Principal component analysis (PCA) of 63 provitamin A maize inbred lines based on population structure when ΔK = 5 (A) and ΔK = 7 (B)

**Figure 4:**
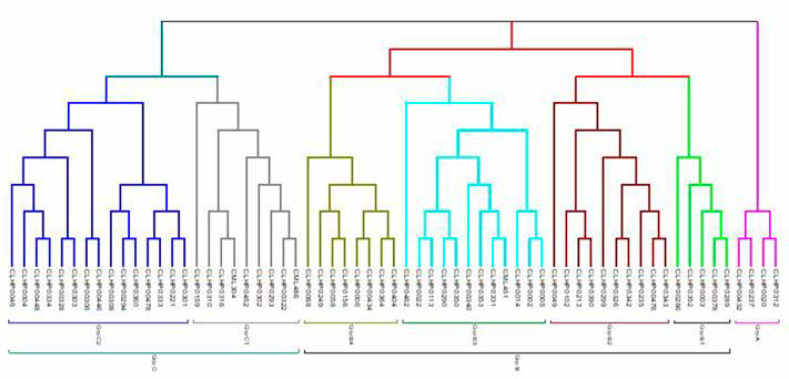
Neighbor-joining tree for 63 inbred lines based on Rogers genetic distance calculated from 137 SNP markers. The subgroups are indicated with different colors. Details of the different subgroups are given in Supplementary Table 1

The results from K = 7 were compared with pedigree and breeding history to characterize the clusters, and it was seen that the STRUCTURE and NJ cluster classifications in many cases placed lines descended from a common ancestor in several groups. For example, lines derived from CML537 were in groups 2 and 4. Two lines in group 4 (entries 46 (CLHP0235) and 47 (CLHP0221)) involved a backcross to CML537 and the donor for carotenoid concentration, while the two lines in group 3 (entries 48 (CLHP0213) and 56 (CLHP0003)) differed from those in group 2 based on the donor for carotenoid concentration. Therefore, the distribution of these lines across the two groups could be attributed to the breeding history in carotenoid conversion program. A similar scenario was applicable to derivatives from line CLQRCWQ109 (entries 17, 32, and 38) that were spread across groups 2, 3 and 5, and for the majority of lines from CML506 (entries 8, 10, 14, 26, and 33) (Table S1). The exceptions were lines derived from CML496 and CML486 that were placed in two separate groups despite the fact that the same donor line for carotenoid concentration was used in each case. Thus, clusters must be assumed to form based on the combined contribution of many parents, and are not formed based on any one ancestral line or phenotypic trait (such as carotenoid concentration). The clusters formed by both NJ dendrogram and STRUCTURE analysis were fairly clearly delineated, but a PCA of these data (first three PCs explained 25.5% of the total SNP variation among the lines for both K = 5 and K = 7) did not show clear separations at all (Fig. 4).

## Discussion

Utilization of diverse sources of provitamin A maize lines with different carotenoid content is important for a new biofortified maize genetic enhancement program. Use of molecular markers to infer genetic diversity in yellow endorsperm and provitamin A maize has been reported in many studies (Menkir et al. 2006; Suwarno et al. 2014; Adeyemo & Omidiji 2014). The number of alleles recorded in this study was higher than that reported in studies with diverse tropical yellow inbred maize lines (Adeyemo and Omidiji 2014; Badu-Apraku et al. 2013; Muthusamy et al. 2014). The average gene diversity recorded in this study was lower than that reported by Muthusamy et al. (2014) in yellow/carotenoid maize and Sserumaga et al. (2014) in tropical white maize but higher than that reported in other studies (Dao et al. 2014; Van Inghelandt et al. 2010). The GD between pairs of inbred lines in this study was smaller than that reported in most other studies (Adeyemo et al. 2011; Menkir et al. 2006; Muthusamy et al. 2014) but comparable to some other studies (Suwarno et al. 2014; Semagn et al. 2012). Large GD estimates (> 0.5) between some of the pairs of provitamin A lines in this set suggests that there is reasonable diversity to choose from, and possibly high levels of heterosis between those pairs with high GD. Molecular marker distance may not predict heterosis accurately in cases where the lines are distantly related (Melchinger et al. 1990; Reif et al. 2003)”, but the pairs with very low distances will probably yield no heterosis, and these crosses can be avoided based on the molecular data. To determine how these exotic provitamin A lines should be classified for use in SSA, they must now be crossed to testers of known SSA maize heterotic groups. For example, the well-known white grain maize testers CML312 (heterotic group A) and CML444 (heterotic group B) that have since been converted to yellow endosperm (CIMMYT, unpublished data) would be some of the suitable testers to use. Suwarno et al. (2014) proposed the use of molecular marker-based GD as way to formulate heterotic groups among provitamin A maize lines developed at CIMMYT.

The relative kinship coefficient reflects the approximate degree of identity between two given individuals (Dao et al. 2014; Hardy and Vekemans 2002), and the lines in this study appear to have a low proportion of common alleles. While the kinship between these lines are higher than in many studies (Wen et al. 2011; Semagn et al. 2012; Wu et al. 2016) the lines in this study were not chosen for diversity, but rather, for good expression of a single trait. This would normally cause a good deal of common alleles, but each line in this study is potentially contributing unique alleles at many of the loci surveyed which should reflect a more diverse genetic pool for breeding provitamin A maize for mid-altitude Africa. The clustering of maize lines based on pedigree or origin is rarely straightforward, unless a group of lines have been selected specifically to be diverse and represent different breeding programs, countries of origin, or growing environment (for example, Suwarno et al. 2015; Lu et al. 2009). Separations based on pedigree or selected traits are rarely found (Warburton et al. 2002; Xia et al. 2005), especially in smaller populations such as the present study. To structure a biofortification breeding program that would utilize this set of provitamin A lines, it will be important to make use of both molecular-based grouping and pedigree of the lines where molecular information does not distinguish lines. In addition, a few representative lines from each of the 7 clusters could be crossed with testers from existing African heterotic groups to help determine which will form the best hybrids; further testcrossing with the most productive heterotic patterns in an efficient and directed manner, and application of the most appropriate selection indices could help identify new productive, adapted provitamin A hybrids. This should allow the use of the molecular data presented here to assist in decision-making regarding the lines to be used for hybrid formation and testing as a short-term objective, and which lines to use for breeding starts as a long-term objective.

In conclusion, there was moderate genetic distance among some of the provitamin A lines used in this study, more than expected due to the common parents used as donor lines for carotenoid content. The results of this study indicated that a combination of molecular marker analyses, pedigree/breeding history, and directed testcrossing is needed to assign inbred lines to sub-groups for breeding decisions. There is substantial genetic variation in this set of provitamin A lines which will be useful for a new maize biofortication program in Uganda.

## Electronic Supplementary Material (ESM)

Electronic Supplementary Material (ESM) associated with this article can be found at the website of CRC at https://akademiai.com/loi/0806

Electronic Supplementary *Table S1*. List of inbred lines used in the study

Electronic Supplementary *Table S2*. List of 137 SNP markers used to genotype the provitamin A lines and summary statistics

Electronic Supplementary *Figure S1*. Distribution of pairwise Roger’s genetic distance calculated for 63 provitamin A maize inbred lines genotyped with 137 SNPs

Electronic Supplementary *Figure S2*. Distribution of pairwise relative kinship calculated for 63 provitamin A maize inbred lines genotyped with 137 SNPs. Relative kinship values close to 0 indicate no relationship
